# Factors influencing health service utilization among 19,869 China’s migrant population: an empirical study based on the Andersen behavioral model

**DOI:** 10.3389/fpubh.2025.1456839

**Published:** 2025-01-23

**Authors:** Yalin Song, Jingru Liu, Shuming Yan, Mingze Ma, Clifford Silver Tarimo, Yiyang Chen, Yanhong Lai, Xinghong Guo, Jian Wu, Beizhu Ye

**Affiliations:** ^1^College of Public Health, Zhengzhou University, Zhengzhou, China; ^2^Department of Science and Laboratory Technology, Dar es Salaam Institute of Technology, Dar es Salaam, Tanzania

**Keywords:** migrant population, health service utilization, Anderson model, lifestyle, public health

## Abstract

**Background:**

With the surge in population migration across China, particularly from rural to urban regions, the migrant population encounters unique challenges in accessing healthcare services. This study addresses the gap in health service utilization among migrants, specifically examining their engagement with basic public health services and the factors that impact these interactions.

**Methods:**

In the year 2023, a cross-sectional survey was carried out among 19,869 migrants in China, employing a multi-stage stratified sampling technique for participant recruitment. The analysis was guided by the Andersen Health Service Utilization Model, examining predisposing, enabling, and needing factors related to health service utilization. Chi-square test and multivariate logistic regression were used to explore the group difference and the associations between demographic characteristics and utilization of health service.

**Results:**

The study revealed significant underutilization of health services among the migrant communities, with only a minority having established health records or engaged with family doctors. Additionally, there existed a notable disparity in health education and the proactive pursuit of health knowledge. Age, gender, educational level, socioeconomic status, employment status, and proximity to healthcare facilities significantly influenced health service utilization. Notably, healthier lifestyle choices correlated with the increased involvement in health services.

**Conclusion:**

The findings highlight a substantial need for targeted health policy interventions and healthcare system reforms to enhance the accessibility and utilization of health services among the migrant population in China. Promoting education on health services, improving healthcare infrastructure, and addressing the unique needs and barriers encountered by migrants are crucial measures for achieving equitable health outcomes.

## 1 Introduction

As China’s socio-economic landscape continues to evolve, there has been a notable trend of inter-provincial and rural-to-urban population migrations. The number of migrants escalated from 102.3 million in 2000 to 247 million in 2015, accounting for 18% of the total population (1–3), primarily comprising rural laborers, migrant workers, and university students. During population migration, labor resources are effectively allocated, facilitating ongoing national social and economic development. However, migrant populations often undergo lifestyle changes and face challenges in areas such as employment, healthcare, housing, and insurance. Compared to the resident population, migrant populations, faced with instability and lacking family support, public infrastructure, and social support networks, are prone to marginalization and exclusion from the local health system (4). This results in limited access to available healthcare resources and often places them at higher risk of poor health outcomes than the local population, including a high prevalence of environmental diseases, infectious diseases, and stress-related disorders (5). These concerns not only profoundly affect their personal and familial lives but also pose considerable challenges to social stability. Given the scale of the migrant population and its role in accelerating modernization and industrialization, a comprehensive understanding of healthcare utilization and its determinants among this population is crucial for enhancing healthcare accessibility, promoting health development, and fostering social integration.

Undeniably, due to the temporary and informal nature of their employment, the migrant population faces numerous barriers in accessing healthcare services. Previous studies have shown that among African immigrant youth in Canada, issues related to regional discrimination, identity and cultural influences adversely affected mental health, yet only 13.3% were willing to seek mental health services (6). A study of Chinese adults aged 45 years and older showed that the prevalence of unmet medical needs was 13.0% and was more concentrated among those with multiple health conditions and mental health problems (7, 8).

In China, basic public health services have been recognized as one of the ‘fundamental rights’ for all. However, due to limitations in the healthcare insurance system, the household registration (hukou) system, and health monitoring systems, migrants face challenges in accessing adequate health services (9–11). The distribution of health resources in China has an inverted pyramid structure, with health resources mainly concentrated in secondary and tertiary health institutions, and with limited resources in grass-roots health institutions, which affects the supply of health services (12). From needs perspective, several studies have shown that the level of ignorance or lack of knowledge about the NBPHSP and participation procedures is higher among the migrant population compared to the local population (13). Only 54.5% of the migrant population met the health education standards in 2017, which falls significantly short of the national goal set by relevant departments for achieving a health education coverage rate of >95% by 2020 (14). A study conducted in 2023 found that 63.33% of the household population in areas where the equalization policy was implemented had a health record, compared to 31.32% of the migrant population. The under-utilization of health services by the migrant population has become an important public health issue (15).

However, although previous studies have identified issues in healthcare utilization among migrant populations, existing research on the factors influencing healthcare use remains limited, lacks systematic exploration, and faces significant constraints. Firstly, many studies have concentrated only on specific subgroups such as adolescent girls (16), the older adults (17), rural populations (10), and ethnic minorities (18), and have not analyzed the migrant population as a whole. Secondly, the selection of health service utilization indicators in some research is restrictive, mostly limiting themselves to one type of health service (19, 20), failing to fully reflect the utilization of basic public health services. Additionally, some studies lack a theoretical analytical framework (21, 22), while others focus solely on health outcomes among migrants, such as hypertension (22), drug-resistant tuberculosis (23), and mental health conditions (24). Therefore, developing effective strategies to examine disparities in health service utilization among the migrant population is crucial.

To our knowledge, no study has comprehensively and systematically analyzed the factors influencing healthcare utilization among migrant populations. This is a critical issue, as without a thorough understanding of the factors affecting healthcare use in this group, health policies and social services may lack a scientific foundation, potentially resulting in indiscriminate and ineffective interventions. Therefore, the primary aim of this study is to systematically examine the key factors influencing healthcare utilization across various sociodemographic and health backgrounds, offering a basis for future policy interventions. The specific objectives of this study are to (i) describe the sociodemographic characteristics, lifestyle factors, and healthcare utilization patterns of the migrant population; (ii) identify key determinants of healthcare utilization among migrants; and (iii) analyze the associations between migrants’ characteristics, lifestyle, and healthcare utilization. The core intent of this study is to contribute to the growing body of literature on healthcare utilization among China’s migrant population. Its significance lies in addressing healthcare access for migrants, a socially vulnerable group, thereby providing a foundation for policies aimed at enhancing healthcare access for this population and offering valuable social application.

### 1.1 Analytic framework

The Andersen Healthcare Utilization Model, based on a social systems approach and validated through decades of empirical research, is widely recognized as a reliable theoretical framework for examining healthcare utilization behaviors (9, 16, 19, 25). Focused on identifying the determinants of healthcare utilization, it considers both social and individual factors from a systems analysis perspective. According to this model, healthcare utilization (including health records, health education, family doctor services, and preventive care knowledge) is driven by three dynamic factors: predisposing, enabling, and need factors (PEN). Predisposing factors include sociodemographic characteristics such as age, race, and gender, while enabling factors involve family support, health insurance, and employment status. The need for healthcare encompasses both perceived and actual needs. This study applies the Andersen Healthcare Utilization Model to conduct a comprehensive survey of healthcare utilization among the migrant population in Henan Province in 2023. Analyzing key factors influencing healthcare utilization from a systems perspective based on the PEN framework, the study’s findings will provide new insights for promoting equitable access to public health services across healthcare institutions in China and in other countries and regions.

## 2 Materials and methods

### 2.1 Data acquisition and study population

The data were collected from August 3 to August 29, 2023, in Henan Province, China. The migrant population in the study refers to the population of Henan origin aged 18 years and above who move across counties and cities within Henan Province or flow into foreign provinces. Given that healthcare decisions for individuals under the age of 18 are typically made by their parents or guardians, minors were excluded from this study.

**Figure 1:**
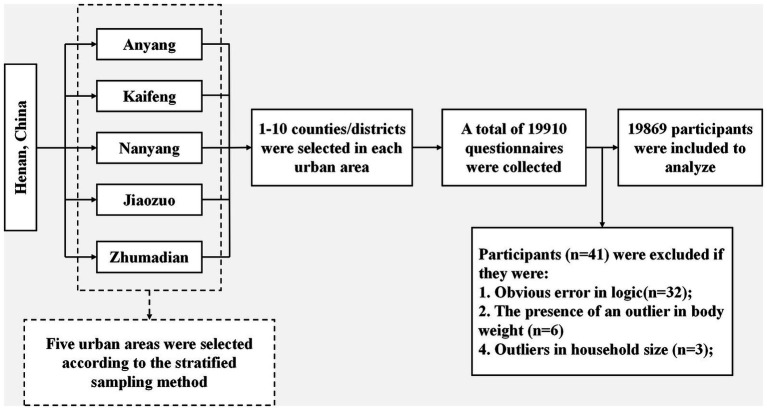
Flow chart of study population sampling.

Using a stratified sampling approach, five urban areas within Henan province were selected in accordance with the study’s feasibility criteria, namely Anyang, Kaifeng, Nanyang, Jiaozuo, and Zhumadian. Subsequently, 1 to 10 counties or districts were randomly selected within each urban area based on the proportion of the migrant population. This was followed by the recruitment of migrant participants for the survey from these selected counties or districts through convenience sampling. All participants in this study participated voluntarily. Inclusion criteria for participants were: (1) being part of the migrant population, (2) voluntary participation, and (3) age 18 years or older. Exclusion criteria included: inability to comprehend the questionnaire questions due to cognitive dysfunction or other illnesses, and inability to independently complete the questionnaire. A total of 19,910 questionnaires were initially collected, and after excluding 41 questionnaires due to missing information or disqualification, 19,869 valid questionnaires were eventually included in the analysis. The response rate was around 99.8%. All data were acquired through online completion using the Questionnaire Star platform. The detailed sample screening process is shown in Figure 1.

The questionnaire was designed with reference to the National Health Services Survey of China and previous researches, encompassing four key areas: predisposing factors (demographic and socio-structural characteristics), enabling factors (personal/family resources and community resources), needing factors (general health status), and health service utilization (such as establishing health records, seeking health knowledge, registering with family doctors, and receiving health education). Details of the questionnaire can be found in Supplementary file 1. Quality assurance measures for this survey included evaluating the questionnaire, training the investigators, and requiring on-site supervisors to oversee the survey process. Before implementation, the questionnaire was reviewed, edited, and validated by experts from health administration departments, clinical teaching hospitals, and community health service institutions. An independent review was conducted to ensure the completeness of the questionnaires and the validity of the collected data. A pilot survey covering 127 individuals was conducted from 2023-07-08 to 2023-07-10 to assess the applicability of the questionnaire and the field survey procedures.

### 2.2 Ethics statement

This cross-sectional investigation examines the determinants of health service utilization within migrant people and has received ethical approval from the Life Sciences Ethics Committee of Zhengzhou University (protocol code ZZUIRB 2024–54). Informed consent was obtained from all participants involved in this study, ensuring the confidentiality of participant information. The research was carried out in alignment with the Declaration of Helsinki, with all methodologies rigorously adhering to established guidelines and regulations.

### 2.3 Variables selected based on the Anderson model

#### 2.3.1 Independent variables

Three types of variables in Anderson’s health service utilization model were selected as independent variables based on the literature. The specific variables and categorization are as follows.

#### 2.3.2 Predisposing factors

- *Age* Ages of the survey respondents in this study spanned a wide range, based on the characteristics of the age distribution of the Chinese population, relevant researches and characteristics of statistical analysis, age was divided into the following categories for analysis and description with ten years of age as a level: (1 = 18–25, 2 = 26–35, 3 = 36–45, 4 = 46–55 and 5 = 56 and above).
- *Gender* was a binary variable (0 = female, 1 = male).
- *Educational level* was a multiclassification variable (1 = primary and below, 2 = junior high school, 3 = high school and university or college degree and above).
- *Ethnicity* was a binary variable (0 = han ethnic, 1 = minorities).
- *Marital status* was a multiclassification variable (1 = unmarried, 2 = married and 3 = divorced/widowed).
- *BMI (kg/m^2^)* as a multiclassification variable was divided into three levels (1 = 18.5 and below, 2 = 28.5–27.9 and 3 = 28 and above).
- *Solitary* was a binary variable (0 = no, 1 = yes).
- *Number of children* was a multiclassification variable (0 = none, 1 = 1, 2 = 2, and 3 = 3+).

#### 2.3.3 Enabling factors

- *Incoming monthly* is based on China’s per capita income level situation and related research (9), and is categorized as follows: (1 = 3,000 RMB and below, 2 = 3,000–4,999 RMB and 3 = 5,000 RMB and above).
- *Employment status* was a multiclassification variable (1 = permanent work, 2 = temporary work and 3 = none).
- *Insurance status* was a binary variable (0 = no, 1 = yes).
- *Housing condition* was a binary variable (0 = own house and 1 = rent).
- *SES* was divided into three levels (1 = low, 2 = moderate and 3 = high).
- *Distance from residence to nearest medical institution* was one of the items in China’s National Assessment of Accessibility to Basic Health Services (NABHS) that were categorized as follows: (1 = 15 min and below, 2 = 15-30 min, 3 = 31-60 min and 4 = 60 min and above).
- *Mental health status* primarily focused on participants’ depression, assessed using the PHQ-9 questionnaire (26), which consists of nine criteria for diagnosing depressive disorder from the DSM-IV. Each item scores ranging from 0 (not at all) to 3 (nearly every day), with a total score range of 0–27. The severity of symptoms can be evaluated by the total score, where PHQ-9 scores are categorized as follows: <5: no depression symptoms, 5–9: mild, 10–14: moderate, 15–19: moderately severe, and ≥ 20: severe depression symptoms. The PHQ-9 has been formally validated through structured diagnostic interviews by mental health professionals, a PHQ-9 score ≥ 10 has a sensitivity and specificity of 88% for detecting major depression. The questionnaire demonstrated high reliability and validity, with a Cronbach’s *α* of 0.931 and a Kaiser-Meyer-Olkin (KMO) value of 0.946.
- *Healthy lifestyle score* Lifestyle was evaluated using a 10-item scale covering aspects such as weight, diet, exercise, sleep, smoking, drinking, stress, relationships, work/study, and health screenings, with scores ranging from 10 to 50. Each item was rated on a 1–5 scale, where higher scores denote healthier lifestyles (27). Scores were divided into quartiles to reflect different health levels. Q1 (first quartile of lifestyle score):10–29, Q2 (second quartile of lifestyle score): 30–33, Q3 (third quartile of lifestyle score): 34–37 and Q4 (fourth quartile of lifestyle score): 38–50. The questionnaire demonstrated high reliability and validity, with a Cronbach’s *α* of 0.749 and a Kaiser-Meyer-Olkin (KMO) value of 0.804.

#### 2.3.4 Needing factors

- *Having chronic disease* was a binary variable (0 = no, 1 = yes).
- *Self-evaluation general health status* was a multiclassification variable (1 = healthy, 2 = general and 3 = unhealthy).
- *Illness in the last two weeks* was a binary variable (0 = no, 1 = yes).
- *Requires hospitalization not hospitalized* was a binary variable (0 = no, 1 = yes). The indicator “needed but not hospitalized” is based on instances where, within the past year, a doctor diagnosed a need for hospitalization which was not pursued.

### 2.4 Utilization of health services

#### 2.4.1 Outcome variables

Utilization of health service was set as outcome variables. The health service utilization currently studied included four categories (health education, health records, family doctor and health knowledge). Based on the utilization or non-utilization of the health service, a dichotomous variable was created for each health service, with an answer of ‘0’ or ‘1’. In terms of the definition of the clusters, for each field of health service, non-utilization was assigned ‘0’ and utilization was assigned ‘1’. There were four types of health service, and participants received one point for each of the four types of health service. Participants were divided into three categories: 0 health service clusters, 1–2 health service clusters and 3–4 health service clusters (28). Details of the points assigned to each health service are as follows:

- *Health education* Participants were inquired whether they had received health education from community health service centers (stations) or township health clinics at their workplace locations in the past year. Responses were formatted as no (coded as 0) vs. yes (coded as 1).
- *Health records* Participants were asked if they had established a resident health record. Responses were formatted as no (coded as 0) vs. yes (coded as 1).
- *Family doctor* Participants were queried on whether they had enrolled in family doctor services. Responses were formatted as no (coded as 0) vs. yes (coded as 1).
- *Health knowledge* Participants were questioned about actively seeking health knowledge or related assistance from the community at their workplace locations within the last year. Responses were formatted as no (coded as 0) vs. yes (coded as 1).

### 2.5 Statistical analysis

**Figure 2:**
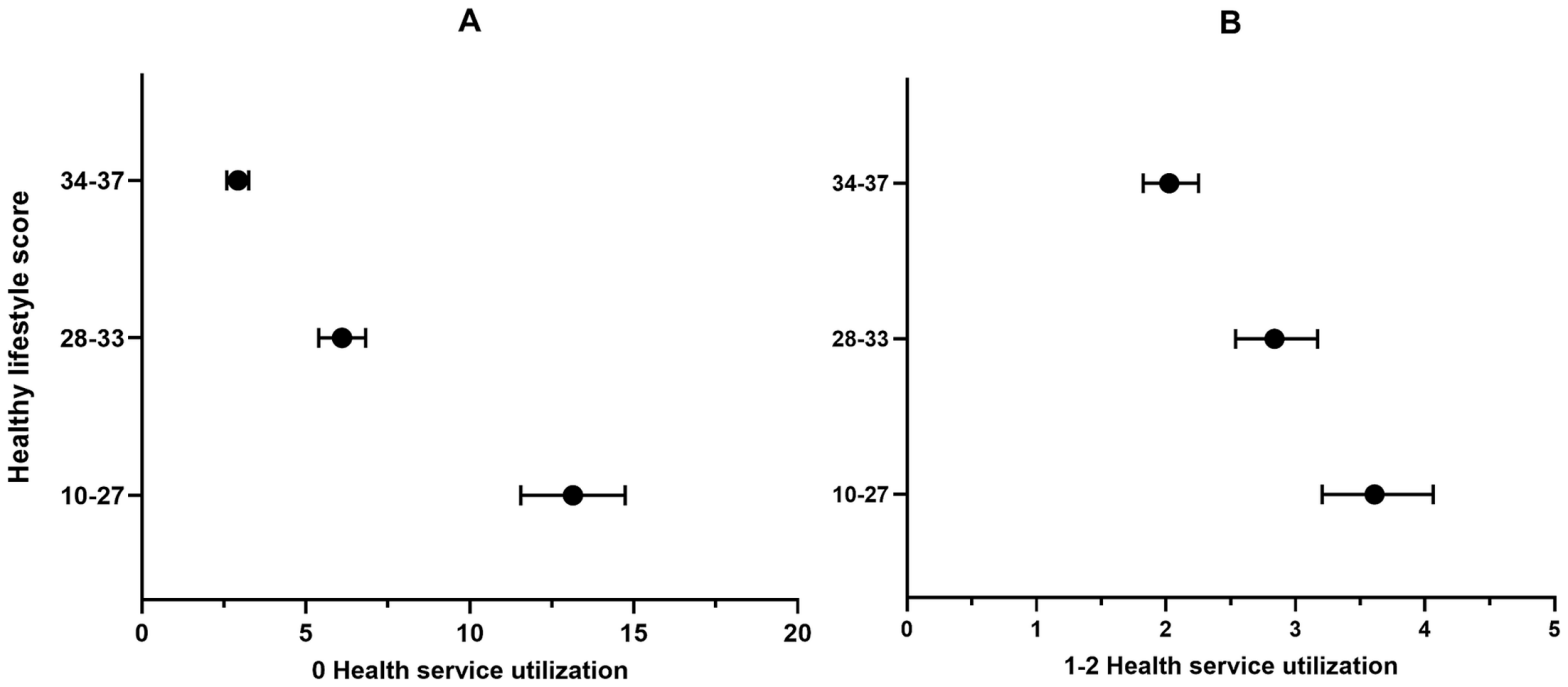
Health service utilization of participants with different lifestyle scores (*n* = 19,869). *p < 0.05, **p < 0.01. Panel A: 0 Health service utilization; Panel B: 1-2 Health service utilization. Q1 (the first quartile): lifestyle scores ranged from 10 to 29; Q2 (the second quartile): lifestyle scores ranged from 30 to 33; Q3 (the third quartile): lifestyle scores ranged from 34 to 37; Q4 (the fourth quartile): lifestyle scores ranged from 38 to 50.

**Figure 3:**
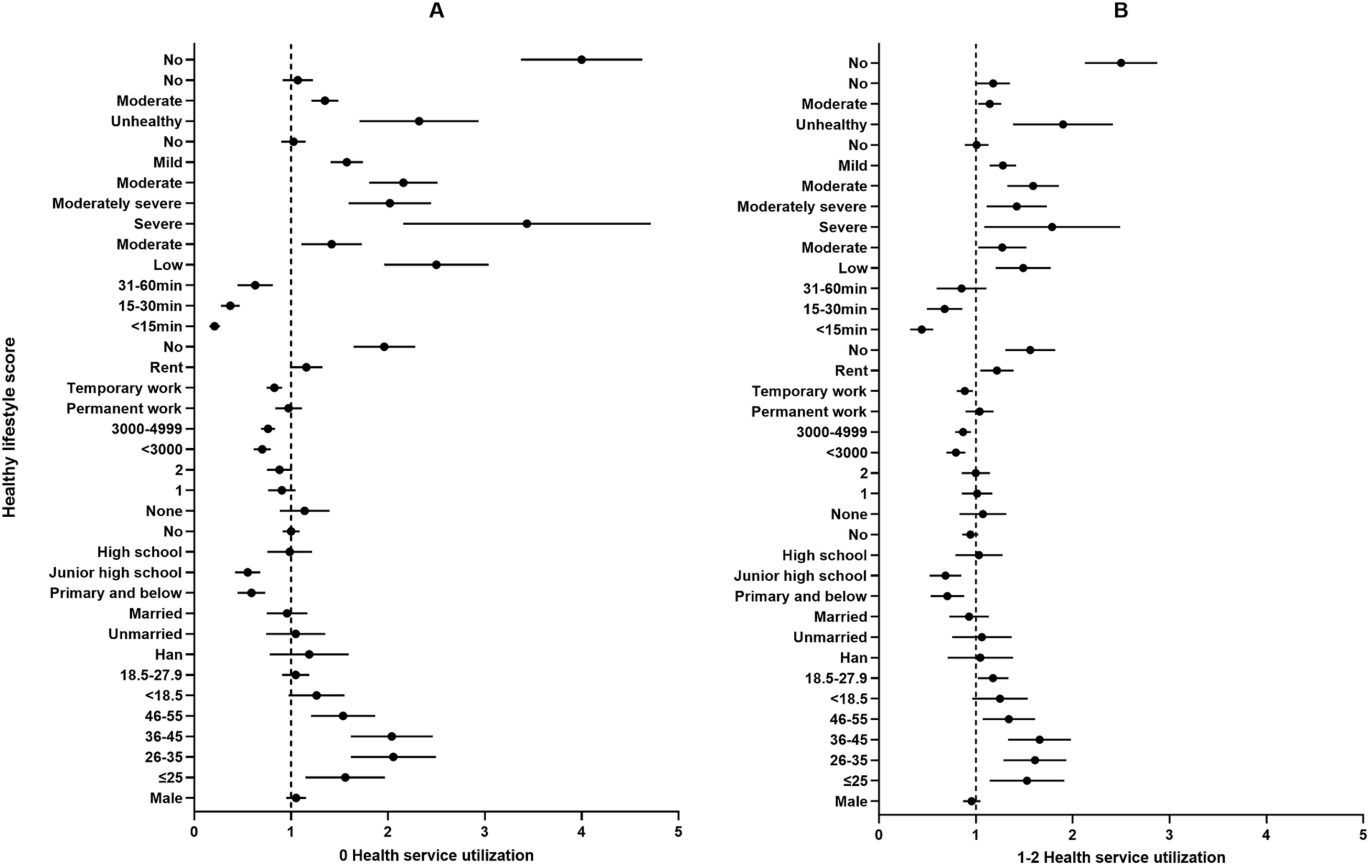
Multiple logistic regression analysis of health service utilization among participants (*n* = 19,869). *p < 0.05, **p < 0.01. Panel A: 0 Health service utilization; Panel B: 1-2 Health service utilization. Incoming refers to the average monthly income in Renminbi (RMB) per person for the year 2022; Distance to healthcare facilities measures the time taken using the most accessible mode of transportation from one’s residence while employed to the nearest medical service institution; SES represents self-assessed socioeconomic status. Mental health status is assessed using the PHQ-9 scale to measure the severity of depressive symptoms, with scores ranging from 0–4 indicating no depression, 5–9 mild depression, 10–14 moderate depression, 15–19 moderately severe depression, and 20–27 severe depression; CHDs denote the presence of chronic diseases diagnosed by a doctor; Physical health status reflects self-assessed health over the past year. Illness in the past two weeks indicates whether one has been sick (injured) or felt unwell during the last two weeks; un-hospitalized despite need represents cases where a doctor recommended hospitalization in the past year, but the individual was not hospitalized. BMI, body mass index; SES, socioeconomic status; CHDs, chronic diseases; PHQ-9, the patient health questionnaire-9.

Firstly, descriptive analyses were conducted to present the frequency (N) and percentage (%) of cases for categorical variables, highlighting sample distributions. Secondly, cross-tabulations and Pearson’s chi-square tests were applied to evaluate significant associations between the Andersen model variables (predisposing, enabling, and need factors) and the utilization of four healthcare services (health records, health education, preventive care knowledge, and family physician services) (Table.1). Thirdly, Pearson’s chi-square tests were also employed to assess clustering patterns across three categories of healthcare service use (0, 1–2, 3–5), examining differences across groups. Finally, multivariate logistic regression analyses were conducted to estimate associations between healthcare service utilization groups and sociodemographic characteristics, identifying potential determinants of healthcare utilization patterns among migrant populations (Figures 2, 3). All analyses were performed using IBM SPSS Statistics Version 24.0 for Windows, with two-sided tests and statistical significance set at *p* < 0.05. Results are reported as odds ratios (OR) with 95% confidence intervals (CI). All missing values were excluded from the statistical analysis.

**Table 1:** Health service utilization of participants with different sociodemographic characteristics (*n* = 19,869).

Variables	Total	Health record		Health care knowledge		Family doctor		Health education	
		Yes	No	Yes	No	Yes	No	Yes	No
	*N* (%)	*N* (%)	*N* (%)	*N* (%)	*N* (%)	*N* (%)	*N* (%)	*N* (%)	*N* (%)
Predisposing									
Gender									
Male	13,418 (67.5)	4,183 (31.2)	9,235 (68.8)	3,115 (23.2)	10,303 (76.8)	2,747 (20.5)	10,671 (79.5)	5,155 (38.4)	8,263 (61.6)
Female	6,451 (32.5)	2,192 (34.0)	4,259 (66.0)	1894 (29.4)	4,557 (70.6)	1,484 (23.0)	4,967 (77.0)	3,100 (48.1)	3,351 (51.9)
χ2		15.727**		87.241**		16.660**		166.566**	
Age (years)	37.066 (9.840)	37.827 (10.400)	36.708 (9.544)	37.479 (10.775)	36.928 (9.501)	38.560 (10.556)	36.663 (9.598)	37.742 (10.381)	36.587 (9.409)
≤ 25	2,652 (13.3)	829 (31.3)	1823 (68.7)	765 (28.8)	1887 (71.2)	508 (19.2)	2,144 (80.8)	1,132 (42.7)	1,520 (57.3)
26–35	6,831 (34.4)	2069 (30.3)	4,762 (69.7)	1,585 (23.2)	5,246 (76.8)	1,329 (19.5)	5,502 (80.5)	2,595 (38.0)	4,236 (62.0)
36–45	6,423 (32.3)	2007 (31.2)	4,416 (68.8)	1,498 (23.3)	4,925 (76.7)	1,280 (19.9)	5,143 (80.1)	2,605 (40.6)	3,818 (59.4)
46–55	3,171 (16.0)	1,118 (35.3)	2053 (64.7)	863 (27.2)	2,308 (72.8)	844 (26.6)	2,327 (73.4)	1,493 (47.1)	1,678 (52.9)
≥56	792 (4.0)	352 (44.4)	440 (55.6)	298 (37.6)	494 (62.4)	270 (34.1)	522 (65.9)	430 (54.3)	362 (45.7)
χ2		83.180**		116.848**		159.141**		132.617**	
BMI (kg/m^2^)	23.843 (3.506)	23.824 (3.426)	23.852 (3.542)	23.670 (3.493)	23.901 (3.508)	23.804 (3.414)	23.853 (3.530)	23.700 (3.428)	23.944 (3.556)
< 18.5	981 (4.9)	282 (28.7)	699 (71.3)	270 (27.5)	711 (72.5)	184 (18.8)	797 (81.2)	411 (41.9)	570 (58.1)
18.5–27.9	16,699 (84.1)	5,430 (32.5)	11,269 (67.5)	4,234 (25.4)	12,465 (74.6)	3,614 (21.6)	13,085 (78.4)	7,019 (42.0)	9,680 (58.0)
≥28	2,189 (11.0)	663 (30.3)	1,526 (69.7)	505 (23.1)	1,684 (76.9)	433 (19.8)	1756 (80.2)	825 (37.7)	1,364 (62.3)
χ2		9.693**		8.287*		7.967*		15.090**	
Ethnicity									
Han	19,602 (98.7)	6,266 (32.0)	13,336 (68.0)	4,921 (25.1)	14,681 (74.9)	161 (21.2)	15,441 (78.8)	8,129 (41.5)	11,473 (58.5)
Minorities	267 (1.3)	109 (40.8)	158 (59.2)	88 (33.0)	179 (67.0)	70 (26.2)	197 (73.8)	126 (47.2)	141 (52.8)
χ2		9.485**		8.618**		3.913*		3.550	
Marital status									
Unmarried	3,615 (18.2)	1,084 (30.0)	2,531 (70.0)	961 (26.6)	2,654 (73.4)	630 (17.4)	2,985 (82.6)	1,446 (40.0)	2,169 (60.0)
Married	15,431 (77.7)	5,050 (32.7)	10,381 (67.3)	3,871 (25.1)	11,560 (74.9)	3,451 (22.4)	11,980 (77.6)	6,512 (42.2)	8,919 (57.8)
Divorced/Widowed	823 (4.1)	241 (29.3)	582 (70.7)	177 (21.5)	646 (78.5)	150 (18.2)	673 (81.8)	297 (36.1)	526 (63.9)
χ2		13.186**		9.730**		47.412**		16.379**	
Educational level									
Primary and below	784 (3.9)	202 (25.8)	582 (74.2)	146 (18.6)	638 (81.4)	147 (18.8)	637 (81.3)	257 (32.8)	527 (67.2)
Junior high school	8,239 (41.5)	2,265 (27.5)	5,974 (72.5)	1,681 (20.4)	6,558 (79.6)	1,454 (17.6)	6,785 (82.4)	3,019 (36.6)	5,220 (63.4)
High school	5,231 (26.3)	1931 (36.9)	3,300 (63.1)	1,576 (30.1)	3,655 (69.9)	1,356 (25.9)	3,875 (74.1)	2,472 (47.3)	2,759 (52.7)
University or college degree and above	5,615 (28.3)	1977 (35.2)	3,638 (64.8)	1,606 (28.6)	4,009 (71.4)	1,274 (22.7)	4,341 (77.3)	2,507 (44.6)	3,108 (55.4)
χ2		175.304**		220.388**		141.767**		198.863**	
Solitary									
Yes	10,834 (54.5)	3,349 (30.9)	7,485 (69.1)	2,515 (23.2)	8,319 (76.8)	2,223 (20.5)	8,611 (79.5)	4,314 (39.8)	6,520 (60.2)
No	9,035 (45.5)	3,026 (33.5)	6,009 (66.5)	2,494 (27.6)	6,541 (72.4)	2008 (22.2)	7,027 (77.8)	3,941 (43.6)	5,094 (56.4)
χ2		15.050**		50.352**		8.555**		29.295**	
Number of children									
None	4,331 (21.8)	1,324 (30.6)	3,007 (69.4)	1,136 (26.2)	3,195 (73.8)	788 (18.2)	3,543 (81.8)	1717 (39.6)	2,614 (60.4)
1	5,004 (25.2)	1,659 (33.2)	3,345 (66.8)	1,337 (26.7)	3,667 (73.3)	1,142 (22.8)	3,862 (77.2)	2,172 (43.4)	2,832 (56.6)
2	8,547 (43.0)	2,786 (32.6)	5,761 (67.4)	2,119 (24.8)	6,428 (75.2)	1902 (22.3)	6,645 (77.7)	3,621 (42.4)	4,926 (57.6)
3 and above	1987 (10.0)	606 (30.5)	1,381 (69.5)	417 (21.0)	1,570 (79.0)	399 (20.1)	1,588 (79.9)	745 (37.5)	1,242 (62.5)
χ2		10.503*		28.018**		38.236**		29.372**	
Enabling									
Incoming monthly (RMB)									
<3,000	3,254 (16.4)	1,066 (32.8)	2,188 (67.2)	913 (28.1)	2,341 (71.9)	804 (24.7)	2,450 (75.3)	1,468 (45.1)	1786 (54.9)
3,000–4,999	7,233 (36.4)	2,332 (32.2)	4,901 (67.8)	1880 (26.0)	5,353 (74.0)	1,594 (22.0)	5,639 (78.0)	3,173 (43.9)	4,060 (56.1)
≥5,000	9,382 (47.2)	2,977 (31.7)	6,405 (68.3)	2,216 (23.6)	7,166 (76.4)	1833 (19.5)	7,549 (80.5)	3,614 (38.5)	5,768 (61.5)
χ2		1.300		28.927**		42.291**		68.479**	
Employment status									
Permanent work	8,214 (41.3)	2,870 (34.9)	5,344 (65.1)	2,200 (26.8)	6,014 (73.2)	1703 (20.7)	6,511 (79.3)	3,520 (42.9)	4,694 (57.1)
Temporary work	9,211 (46.4)	2,800 (30.4)	6,411 (69.6)	2,215 (24.0)	6,996 (76.0)	2051 (22.3)	7,160 (77.7)	3,765 (40.9)	5,446 (59.1)
None	2,444 (12.3)	705 (28.8)	1739 (71.2)	594 (24.3)	1850 (75.7)	477 (19.5)	1967 (80.5)	970 (39.7)	1,474 (60.3)
χ2		54.522**		18.454**		11.348**		10.96**	
Housing condition									
Rent	18,214 (91.7)	5,719 (31.4)	12,495 (68.6)	4,452 (24.4)	13,762 (75.6)	3,820 (21.0)	14,394 (79.0)	7,451 (40.9)	10,763 (59.1)
Own house	1,655 (8.3)	656 (39.6)	999 (60.4)	557 (33.7)	1,098 (66.3)	411 (24.8)	1,244 (75.2)	804 (48.6)	851 (51.4)
χ2		47.256**		68.296**		13.494**		36.770**	
Insurance status									
Yes	17,812 (89.6)	5,981 (33.6)	11,831 (66.4)	4,619 (25.9)	13,193 (74.1)	4,016 (22.5)	13,796 (77.5)	7,630 (42.8)	10,182 (57.2)
No	2057 (10.4)	394 (19.2)	1,663 (80.8)	390 (19.0)	1,667 (81.0)	215 (10.5)	1842 (89.5)	625 (30.4)	1,432 (69.6)
χ2		176.074**		47.545**		160.943**		117.739**	
Distance from residence to nearest medical institution									
< 15 min	9,561 (48.1)	3,893 (40.7)	5,668 (59.3)	3,047 (31.9)	6,514 (68.1)	2,568 (26.9)	6,993 (73.1)	4,650 (48.6)	4,911 (51.4)
15-30 min	7,009 (35.3)	1894 (27.0)	5,115 (73.0)	1,503 (21.4)	5,506 (78.6)	1,252 (17.9)	5,757 (82.1)	2,793 (39.8)	4,216 (60.2)
31-60 min	2,239 (11.3)	426 (19.0)	1813 (81.0)	353 (15.8)	1886 (84.2)	301 (13.4)	1938 (86.6)	611 (27.3)	1,628 (72.7)
> 60 min	1,060 (5.3)	162 (15.3)	898 (84.7)	106 (10.0)	954 (90.0)	110 (10.4)	950 (89.6)	201 (19.0)	859 (81.0)
χ2		721.956**		513.560**		383.619**		616.175**	
SES									
Low	13,627 (68.6)	3,779 (27.7)	9,848 (72.3)	2,838 (20.8)	10,789 (79.2)	2,505 (18.4)	11,122 (81.6)	4,880 (35.8)	8,747 (64.2)
Moderate	5,556 (28.0)	2,268 (40.8)	3,288 (59.2)	1869 (33.6)	3,687 (66.4)	1,490 (26.8)	4,066 (73.2)	2,961 (53.3)	2,595 (46.7)
High	686 (3.5)	328 (47.8)	358 (52.2)	302 (44.0)	384 (56.0)	236 (34.4)	450 (65.6)	414 (60.3)	272 (39.7)
χ2		390.970**		477.041**		240.400**		600.150**	
Mental health status	5.472 (6.053)	3.683 (5.112)	6.317 (6.275)	3.612 (5.114)	6.098 (6.214)	3.782 (5.207)	5.929 (6.183)	3.832 (5.133)	6.637 (6.378)
Normal	10,589 (53.3)	4,287 (40.5)	6,302 (59.5)	3,412 (32.2)	7,177 (67.8)	2,803 (26.5)	7,786 (73.5)	5,421 (51.2)	5,168 (48.8)
Mild	5,055 (25.4)	1,303 (25.8)	3,752 (74.2)	998 (19.7)	4,057 (80.3)	887 (17.5)	4,168 (82.5)	1783 (35.3)	3,272 (64.7)
Moderate	2,301 (11.6)	474 (20.6)	1827 (79.4)	348 (15.1)	1953 (84.9)	313 (13.6)	1988 (86.4)	627 (27.2)	1,674 (72.8)
Moderately severe	1,333 (6.7)	235 (17.6)	1,098 (82.4)	198 (14.9)	1,135 (85.1)	176 (13.2)	1,157 (86.8)	332 (24.9)	1,001 (75.1)
Severe	591 (3.0)	76 (12.9)	515 (87.1)	53 (9.0)	538 (91.0)	52 (8.8)	539 (91.2)	92 (15.6)	499 (84.4)
χ2		802.609**		638.948**		400.005**		997.743**	
Healthy lifestyle score	33.490 (6.217)	35.917 (6.306)	32.343 (5.834)	36.639 (6.296)	32.429 (5.819)	36.040 (6.404)	32.800 (5.982)	35.818 (6.108)	31.835 (5.747)
10–29	6,475 (32.6)	1,275 (19.7)	5,200 (80.3)	822 (12.7)	5,653 (87.3)	835 (12.9)	5,640 (87.1)	1,591 (24.6)	4,884 (75.4)
30–33	5,243 (26.4)	1,455 (27.8)	3,788 (72.2)	1,083 (20.7)	4,160 (79.3)	964 (18.4)	4,279 (81.6)	1988 (37.9)	3,255 (62.1)
34–37	4,724 (23.8)	1760 (37.3)	2,964 (62.7)	1,423 (30.1)	3,301 (69.9)	1,137 (24.1)	3,587 (75.9)	2,372 (50.2)	2,352 (49.8)
38–50	3,427 (17.2)	1885 (55.0)	1,542 (45.0)	1,681 (49.1)	1746 (50.9)	1,295 (37.8)	2,132 (62.2)	2,304 (67.2)	1,123 (32.8)
χ2		1385.746**		1689.180**		876.923**		1873.666**	
Needing									
Having chronic disease									
Yes	3,535 (17.8)	991 (28.0)	2,544 (72.0)	775 (21.9)	2,760 (78.1)	733 (20.7)	2,802 (79.3)	1,255 (35.5)	2,280 (64.5)
No	16,334 (82.2)	5,384 (33.0)	10,950 (67.0)	4,234 (25.9)	12,100 (74.1)	3,498 (21.4)	12,836 (78.6)	7,000 (42.9)	9,334 (57.1)
χ2		32.387**		24.633**		0.802		64.702**	
Self-evaluation general health status									
Unhealthy	1,058 (5.3)	178 (16.8)	880 (83.2)	130 (12.3)	928 (87.7)	132 (12.5)	926 (87.5)	229 (21.6)	829 (78.4)
Moderate	6,775 (34.1)	1,599 (23.6)	5,176 (76.4)	1,144 (16.9)	5,631 (83.1)	1,094 (16.1)	5,681 (83.9)	2,150 (31.7)	4,625 (68.3)
Healthy	12,036 (60.6)	4,598 (38.2)	7,438 (61.8)	3,735 (31.0)	8,301 (69.0)	3,005 (25.0)	9,031 (75.0)	5,876 (48.8)	6,160 (51.2)
χ2		543.522**		559.074**		253.017**		703.356**	
Illness in the last two weeks									
Yes	2,184 (11.0)	618 (28.3)	1,566 (71.7)	610 (27.9)	1,574 (72.1)	516 (23.6)	1,668 (76.4)	841 (38.5)	1,343 (61.5)
No	17,685 (89.0)	5,757 (32.6)	11,928 (67.4)	4,399 (24.9)	13,286 (75.1)	3,715 (21.0)	13,970 (79.0)	7,414 (41.9)	10,271 (58.1)
χ2		16.161**		9.630**		7.961**		9.336**	
Requires hospitalization not hospitalized									
Yes	1,594 (8.0)	632 (39.6)	962 (60.4)	697 (43.7)	897 (56.3)	546 (34.3)	1,048 (65.7)	818 (51.3)	776 (48.7)
No	18,275 (92.0)	5,743 (31.4)	12,532 (68.6)	4,312 (23.6)	13,963 (76.4)	3,685 (20.2)	14,590 (79.8)	7,437 (40.7)	10,838 (59.3)
χ2		45.497**		315.137**		173.650**		68.120**	

**p < 0.01, *p < 0.05.

## 3 Results

### 3.1 Participant characteristics

A total of 19,869 participants were included in this analysis. 67.5% were male, the average age of the participants was 37.1 years, and 34.4% of participants were aged between 26 and 35 years, with an average Body Mass Index (BMI) of 23.843 and 84.1% having a Body Mass Index (BMI) ranging from 18.5 to 27.9. A substantial majority (98.7%) identified as Han ethnicity. Married individuals constituted 77.7% of the sample, and those with a junior high school education accounted for 41.5%. Over half of the participants (54.5%) lived alone, and 43.0% had two children. Regarding economic status, 47.2% reported a monthly income of 5,000 RMB or more. Meanwhile, 41.3% had stable employment. A significant majority (91.7%) were renters, with 89.6% having insurance coverage. Approximately 35.5% of participants could reach the nearest medical facility within 15–30 min. A notable 68.6% perceived their socio-economic status (SES) as low. The average mental health score of the participants was 5.472, more than half of the participants (53.3%) showed no symptoms of depression. Participants’ lifestyle score was 33.490, and 32.6% scored between 10 and 29 on a lifestyle assessment scale. Health perceptions were generally positive, with 60.6% considering themselves to be in good health. Furthermore, 82.2, 89.1, and 92.0% reported no chronic diseases, no illness in the past two weeks, and no instances of needing but not receiving hospitalization based on a doctor’s diagnosis, respectively (Table 1).

### 3.2 Utilization of health services

#### 3.2.1 Health record status

Overall, 67.9% of participants had not established a resident health record. We observed that the proportion of individuals without a health record was comparatively lower among men (68.8%), those aged 26–35 years (69.7%), individuals with a BMI below 18.5 (69.7%), Han ethnicity (68.0%), those who are divorced or widowed (70.7%), those with a primary and below education (74.2%), individuals living alone (69.1%), and those with three or more children (69.5%). Among participants without employment, 71.2% lacked a health record, and this figure rose to 80.8% among those without insurance. Compared to homeowners, renters exhibited a higher proportion without health records (68.6% vs. 60.4%). Participants who required more than 60 min to reach the nearest medical facility, those with a low SES, those experiencing severe depressive states, individuals scoring between 10 and 29 on a lifestyle scale, and those perceiving their health as poor were most likely not to have established a health record, with rates of 84.7, 72.3, 87.1, 80.3, and 83.2%, respectively. In the subset of the population more likely to have a health record, they typically had no chronic diseases and had not been ill in the past two weeks. Among participants who had been diagnosed by a doctor as needing hospitalization but had not been hospitalized, only 39.6% had established the health record (*p* < 0.05 for all) (Table 1).

#### 3.2.2 Health care knowledge

In the past year, only a minority of participants (23.2%) actively sought health care knowledge. This inclination was predominantly observed among women (29.4%), individuals aged 56 and above (37.6%), those not living alone (27.6%), employed participants (26.8%), homeowners (33.7%), and those with insurance (25.9%). The propensity to seek healthcare knowledge tended to decrease with an increase in BMI. Individuals with a high school education were the most likely to seek out health information, while those with primary education or below were the least likely (30.1% vs. 18.6%). As income levels increased, the proportion of individuals seeking health care knowledge declined. Participants were more inclined to seek healthcare knowledge as their self-assessed SES increased, the distance to the nearest medical facility decreased, symptoms of depression lessened, lifestyle scores improved, and their self-assessed health status improved. Moreover, those without chronic diseases (25.9% vs. 21.9%), who had been ill in the past two weeks prior to this study (27.9% vs. 24.9%), and who had been diagnosed by a doctor as needing hospitalization but had not been hospitalized (43.7% vs. 23.6%) were relatively more likely to seek health information (*p* < 0.05 for all) (Table 1).

#### 3.2.3 Family doctor

Over three-quarters (78.7%) of the participants had not registered with a family doctor. Women (23.0%), individuals with a BMI between 18.5 and 27.9 (21.6%), those who were married (22.4%), and participants with high school education (25.9%) exhibited a higher propensity for registering with family doctors. The likelihood of engaging with family medicine services was observed to increase with age, proximity to the nearest medical facility, higher self-assessed SES, better mental health, higher lifestyle scores, and better self-rated health status. Conversely, participants with higher monthly incomes were less likely to register with a family doctor. The data revealed that insured participants were significantly more likely to register for family doctor services (22.5%) compared to those without insurance (10.5%). Among those who had not been ill in the past two weeks and those who had not been diagnosed by a doctor as needing but not receiving hospitalization in the past year, the rates of registering with a family doctor were 79.0 and 79.8%, respectively (*p* < 0.05 for all) (Table 1).

#### 3.2.4 Health education

The findings indicate that more than half (58.5%) of the participants had no history of receiving any health education in the past year. Among those without insurance, having three or more children, with a BMI of 28 or higher, without work, and living in rented accommodation, the proportions who had not received health education in the last year were 69.6, 62.5, 62.3, 60.3, and 59.1%, respectively. Factors such as increased distance to the nearest medical facility, lower self-assessed SES, intensified symptoms of depression, reduced lifestyle scores, and worse self-rated health condition were likely contributors to the decreased likelihood of receiving health education. Additionally, populations more likely to have received health education included those without chronic diseases, those who had not been ill in the past two weeks, and those who had been diagnosed by a doctor as needing hospitalization within the last year but who were not hospitalized (*p* < 0.05 for all) (Table 1).

### 3.3 Association of healthy lifestyle score with health service utilization

Figure 2 illustrates the association between the number of health services utilized and the health lifestyle scores. As the health lifestyle scores decrease, the risk of utilizing 0 health services significantly increases, with the Q1 (OR = 13.080, 95% CI: 11.583–14.771), Q2 (OR = 6.074, 95% CI: 5.400–6.832), Q3 (OR = 2.910, 95% CI: 2.593–3.266) (*p* < 0.05 for all). A similar pattern emerges among subgroups utilizing 1–2 health services, with Q1 presenting an OR of 3.610 (95% CI: 3.207–4.063), Q2 an OR of 2.837 (95% CI: 2.538–3.171), and Q3 an OR of 2.025 (95% CI: 1.823–2.251) (*p* < 0.05 for all).

Figure 3 elucidates the relationship between health service utilization and different demographic. Compared to those aged 56 and above, participants under the age of 56 were identified as having a higher risk of utilizing 0 and 1–2 health services. Notably, within the 18–45 age group, the risk was observed to increase with age, with the 36–45 age group exhibiting the highest risk, indicated by OR of 2.009 (95% CI: 1.630–2.476) for 0 services and 1.636 (95% CI: 1.344–1.991) for 1–2 services. In terms of educational attainment, having a primary school education or less (OR = 1.889, 95% CI: 1.526–2.338) and junior high school education (OR = 1.742, 95% CI: 1.587–1.912) were positively correlated with the utilization of 0 and 1–2 health services. Compared to participants with a monthly income of over 5,000 RMB, those with lower income levels reported a reduced risk of utilizing fewer health services. Participants with permanent (OR = 0.820, 95% CI: 0.726–0.928) and temporary (OR = 0.880, 95% CI: 0.779–0.994) work were less likely to use 0 health service. Concurrently, participants without insurance (OR = 1.944, 95% CI: 1.653–2.288; OR = 1.547, 95% CI: 1.312–1.825) and those who had not been diagnosed by a doctor as needing but not receiving hospitalization in the past year (OR = 3.966, 95% CI: 3.390–4.640; OR = 2.481, 95% CI: 2.136–2.881) exhibited a decreased likelihood of seeking health services. Relative to participants with self-assessed good health status, those with moderate (OR = 1.344, 95% CI: 1.214–1.489; OR = 1.138, 95% CI: 1.026–1.262) and poor health conditions (OR = 2.267, 95% CI: 1.736–2.960; OR = 1.853, 95% CI: 1.409–2.436) showed reduced chances of utilizing 0 and 1–2 health services. Moreover, compared to individuals without depressive symptoms, those with severe, moderately severe, moderate, and mild symptoms had a 3.276 (OR = 2.267, 95% CI: 1.736–2.960), 1.989 (OR = 1.989, 95% CI: 1.610–2.458), 2.139 (OR = 2.139, 95% CI: 1.816–2.520), and 1.568 (OR = 1.568, 95% CI: 1.410–1.744) times higher likelihood, respectively, of not seeking health services (*p* < 0.05 for all).

## 4 Discussions

This study aimed to describe the health service utilization behavior of the migrant population in China through the Anderson Health Service Utilization Model. We investigated several aspects, including the creation of health records, engagement with family doctors, proactive health knowledge seeking, and participation in health education within this demographic. Moreover, we identified variations in health service utilization among the floating population, attributed to various predisposing, enabling, and need factors, as well as lifestyle choices. The primary aim being to enhance the access and use of health services among the floating population.

Overall, due to the unique identity of the floating population, the utilization of basic public health services by China’s floating population remains significantly low and varies across different health services (29). Only 21.3% have engaged in contractual agreements with family doctors, surpassing the findings from the 2018 national survey but falling short of the 30% target set by the “Guidance on Promoting Family Doctor Contract Services” (19). Furthermore, merely 30% of this group reported to have created health records (30), a figure that corroborates earlier research findings (19). Due to the large floating population and unstable living conditions, there are many obstacles to signing up with family doctors and establishing health records (31). The current study revealed that 41.5% of the floating population engaged in health education, marking the highest engagement rate among the four basic public health services evaluated. However, this statistic falls considerably short of the health education coverage goal of over 95% for the domestic floating population outlined in the “13th Five-Year Plan” (32). Furthermore, merely 25.2% of the floating population proactively sought health care information over the last year. Engaging in health education and actively seeking health care information are crucial for comprehending medical data. The socio-economic circumstances of the migrant population’s life and work often restrict their information access, leading to a deficient understanding of their health status and diminishing the availability of health services (33, 34).

Utilizing the Anderson model for health service utilization as a framework, this study identifies both commonalities and variations in the factors affecting the use of four fundamental public health services. In terms of predisposing factors, women, in comparison to men, demonstrated a higher engagement across all four service types. In the context of the growing migrant movement, female migrants represent a notable segment, exhibiting heightened demands for quality of life, health services, and the acquisition of relevant knowledge and information (35). Age-wise, the 26–35 age group displays the least interaction with health services, whereas those aged 56 and older indicated the greatest level of utilization. This pattern may be attributed to the relatively superior health status of younger migrants, who have diminished requirements for health services relative to their elder counterparts. The research indicates that migrants holding a high school diploma are the most active users of health services, while those with only elementary education or less are the least likely to maintain health records, seek healthcare knowledge, or participate in health education. This observation is consistent with previous studies, suggesting that lower educational attainment may be associated with reduced health awareness and a limited understanding of available health services (36, 37). Additionally, the findings show that individuals living with others access health services more frequently, echoing similar findings from other research (19, 38), likely due to a heightened sense of familial duty and a greater pursuit of quality of life among these individuals. Family-based migration is a prevalent phenomenon within the migrant population. Following the enactment of the “universal two-child” policy in China in 2015, a significant number of families have welcomed a second child (39), subsequently elevating the demand for public services, including healthcare, childcare, and education (40). The study reveals a greater propensity among migrants with two children to engage with health services, in contrast to those with three or more children, who demonstrate the lowest engagement levels. This trend may stem from the financial pressures faced by larger families, particularly the increased costs associated with education, which escalate total household expenses and limit discretionary spending, thus impacting the utilization of health services.

Among enabling factors, stable employment significantly increases health service utilization among the floating population, surpassing those in temporary or non-employment situations. Furthermore, homeownership and a self-assessed high socioeconomic status are positively associated with increased health service usage, consistent with existing research (30). This improvement is attributed to the stability of economic resources and better living conditions. The coverage of medical insurance in China has been achieved the rate of approximately 95% in recent years (41), tailored to meet the evolving medical needs of residents and enhance their quality of life. Access to medical insurance plays a crucial role in the health service utilization of the floating population, with insured individuals demonstrating a higher propensity to use health services (9, 19, 20). However, underutilization remains a concern, as less than half of the insured floating population engaged with health services. Undoubtedly, the health insurance system has increased the utilization of basic health service, as well as outpatient and inpatient service, and prevented catastrophic health care payments for vulnerable groups in China, but it is not useful for the migrant population (42). In most countries, the migrant labor force in the majority of industries works without a contract, which often prevents the privilege of enjoying typical national or local health insurance, support service, or social assistance (43). In addition, limited awareness of health care or complex reimbursement procedures increase barriers to accessing health services among migrant population. In addition, limited awareness of health care or complex reimbursement procedures increase barriers to accessing health services among migrant population. A 2018 survey revealed an increase in the accessibility of medical facilities in rural western areas (44), yet only 48.1% of the floating population reported being able to reach the nearest medical facility within 15 min, underscoring significant access barriers compared to the broader population. Additionally, we correlated the distance to the nearest health institution with health service utilization, underscoring the influence of proximity on health service utilization, closer proximity to medical institutions correlates with increased service usage. These findings suggest a need for improved primary healthcare infrastructure and a strengthened public health system.

Lifestyle exerts a significant and enduring influence on personal health, with unhealthy habits contributing to mental health challenges such as depression and anxiety, while also elevating the risk of chronic diseases. The “Healthy China 2030” initiative underscores the importance of healthy behaviors—such as maintaining a balanced diet, engaging in regular physical activity, and limiting tobacco and alcohol use—in bolstering physical well-being (45). This study supports existing literature by demonstrating that different dimensions of lifestyle play an important role in health service utilization, and also further validates the positive impact of a healthy lifestyle on greater participation in health services among migrant population. Awareness of health-related issues typically leads to enhanced utilization and understanding of available health services, fostering a virtuous cycle of health consciousness and the adoption of self-directed health care practices. Consequently, the promotion of healthy lifestyles, coupled with the enhancement of public health literacy, plays a pivotal role in facilitating access to and the effective use of health services.

The findings of this study demonstrate that among the needing factors, both the floating population without chronic diseases and those who perceive themselves as healthy are more likely to utilize health services. Historical surveys have documented that in Western countries, where comprehensive medical strategies have been extensively adopted since the twentieth century (46–48), there has been a steady enhancement in public health standards. This evidence supports the efficacy of initiatives aimed at boosting physical fitness, offering valuable insights for China’s health policy framework. Common barriers to hospitalization, despite apparent need, typically involve financial constraints, a lack of perceived necessity, and time shortages. The recent adoption of a tiered diagnosis and treatment model has notably improved access to health services, leading to a significant reduction in the percentage of urban and rural residents unable to afford hospitalization—from 18.3 and 24.5% in 1998 to 9.0 and 10.2% in 2018 (44), respectively. This study found that only 8.0% of the floating population reported unmet hospitalization needs, highlighting a growing satisfaction with medical service demand as medical security and health service infrastructure improve. However, it’s concerning that utilization rates for essential health services among the floating population requiring hospitalization remain under 50%. This demographic, crucial to the actualization of national basic public health service standards, continues to grapple with challenges related to service awareness and engagement levels.

In general, the results of this study suggest that the Chinese Government should pay attention to the migrant population in all regions in its future efforts to equalize basic public health service. Firstly, in response to the underutilization of health services among migrant population, the capacity of urban and rural community health service providers should be strengthened, and health service projects for migrant population should be actively promoted in order to improve the professionalism, accessibility and convenience of service. Secondly, At the community level, we should strengthen the publicity of health service among migrant population through various channels, so as to raise the overall awareness level of the migrant population, cultivate positive health concepts, and enhance their knowledge, acceptance and participation in the health service model.

### 4.1 Implications of the findings for future studies

Further research should focus on the healthcare needs of migrants across diverse sociodemographic contexts, examining their health risks, healthcare utilization patterns, and demand for medical resources to provide robust data that can inform policy development. Such studies should highlight the shortcomings of the current healthcare system in addressing the needs of migrant populations, particularly in areas such as chronic conditions, infectious diseases, and mental health, as well as reveal how urban–rural disparities, gender differences, and cultural factors influence healthcare needs, offering valuable reference for the design of more equitable policies. Moreover, future research should prioritize strengthening intersectoral collaboration, particularly between health, social security, education, and labor sectors, to address challenges such as the unequal distribution of medical resources, insufficient health education, and barriers in cross-cultural communication. This will help enhance the overall health outcomes of migrant populations. Additionally, research should explore the development of effective intersectoral mechanisms that integrate resources to meet the diverse healthcare needs of migrants. Finally, it is essential to investigate how health considerations can be incorporated into social integration policies for migrants, by designing inclusive health education initiatives, improving health literacy, ensuring equitable access to healthcare, and leveraging legal measures to remove existing policy barriers.

## 5 Strengthens and limitations

Our study has several notable strengths. Firstly, it employed a large cross-sectional study design with a large sample size, which includes the floating population from five cities in Henan Province, covering a wide geographic range across the nation. This breadth ensures a high degree of representativeness, mirroring the broader patterns of health service utilization among China’s migrant population. Secondly, using Anderson’s health service utilization model not only provides a systematic and comprehensive understanding of the basic characteristics of China’s migrant population, but also allows for a comprehensive analysis of the determinants that influence the utilization of basic public health services in this population, as well as providing strong support for targeted interventions. Thirdly, our study focuses on the health service utilization of migrants, a socially disadvantaged group, and provides a basis for policy development to improve their health service utilization and health status, which has significant social application value.

However, there are some limitations to our study. (1) The cross-sectional nature of the study was not sufficient to infer causality. This highlights the need for future cohort studies to more robustly explore healthcare utilization patterns and causality among migrant populations. (2) Self-reported and retrospective data may lead to recall bias, resulting in underreporting of health status and health service utilization information. (3) Endogeneity of health service utilization among the mobile population, such as health status or socio-economic status, may affect the robustness of the findings, and appropriate econometric methods, such as tool variable analysis or propensity score matching, need to be considered in the future to alleviate endogeneity concerns. (4) Health service utilization is a socio-disciplinary issue, and the factors influencing it may not act alone, but rather as a result of synergistic multifactorial effects, which needs to be further investigated. (5) Although the data for this study were collected three years after the COVID-19 pandemic and the survey was not directly affected by it, it is undeniable that the post-pandemic era may change the health service utilization patterns of the migrant population, which in turn may affect the results of this study, and thus caution needs to be exercised when citing the results.

## 6 Conclusion

This study enhances our comprehension of the health status and service utilization by China’s migrant population in 2023, including the determinants of such utilization. Our analysis reveals that overall engagement with health services among this group is limited, with significant variations based on demographic factors. There’s a critical emphasis on addressing the needs of male migrants, individuals with lower education, economic challenges, unemployment, and compromised health. Promoting equity in health services is crucial for achieving broader health equity. Despite their marginal status, the floating population deserves access to full healthcare services. This research highlights the need for healthcare system reforms and improvements in health service infrastructure. It urges policymakers to refine medical resource distribution, amplify targeted public education, and boost health consciousness within the floating population. Future inquiries should delve deeper into the influencers of health service engagement among this demographic, aiming to foster their acceptance of essential public health interventions and ensure equitable access to primary healthcare.

## Data Availability

The raw data supporting the conclusions of this article will be made available by the authors, without undue reservation.
