# Supplementary material for: Factors influencing health service utilization among 19,869 China’s migrant population: an empirical study based on the Andersen behavioral model
Source: Front Public Health. 2025 Jan 23;13:1456839. doi: 10.3389/fpubh.2025.1456839 (PMC11798976; doi:10.3389/fpubh.2025.1456839)
Supplement: Supplementary file 1 [file Supplementary_file_1.docx]

**Survey on employment and health service utilization by migrant workers**

Dear Madam/Sir:

Greetings! Thank you very much for taking time out of your busy schedule to complete this questionnaire. We are conducting this survey in order to find out more about your working life and health service utilization. There is no standard answer to each question, just tell us what you really think and how you are doing. The content of the survey will only be used for statistical and analytical research, we will strictly follow the requirements of the relevant provisions of the Statistical Law of the People's Republic of China, all the results of the survey will not be disclosed to the public, and your personal information will be strictly protected, thank you for your support and cooperation.

**I. Basic information**

| 1.Your gender: 1 Male 2 Female |
| --- |
| 2.Your age： years；Height： cm；weight： kg |
| 3. Your Ethnicity：1 Han 2 Minority |
| 4. Your marital status: 1 Unmarried 2 Married 3 Divorced 4 Widowed |
| 5. What is your level of education?  1 Primary and below 2 Junior high school 3 Senior high school (including secondary school and technical school) 4 College/undergraduate and above |
| 6. What is your type of occupation?  1 Workers in transport, construction, production, manufacturing, etc.  2 People in agriculture, forestry, animal husbandry, fisheries, water production, etc.  3 Individual operators  4 Service sector (service workers, delivery workers, couriers, etc.)  5 Professional and technical staff (medical workers, accountants, teachers, researchers, etc.)  6 Heads of State organs, party organizations, enterprises and institutions  7 No fixed occupation  8 Other |
| 7. Your domicile is: (drop-down box option) |
| 8. Where did you work outside the home in the past year:  Province Municipality (region) District (county, county-level city) |
| 9. In 2022, your average personal monthly income will be approximately _____RMB |
| 10. How many people are in your family (who usually live together)? people  What was the total income of the family last year? _____ten thousand RMB |
| 11. Who are the family members you live with? (Multiple choices possible)  1 Living alone 2 Spouse 3 Child 4 Parent 5 Son-in-law/daughter-in-law 6 Parents-in-law 7 Grandparent(s) 8 Grandchildren 9 Siblings 10 Others |
| 12. How many children do you currently have?  1 None 2 One 3 Two 4 Three and more |
| 13. Do you plan to have children in the next two years?  1 Yes (skip to Q15) 2 No 3 Don't think so (skip to Q15) |
| 14. What is the main reason why you do not plan to have children in the next two years? (Choose up to 3)  1 The cost of childbearing and child rearing is high and the financial burden is heavy  2 After giving birth, parents are busy at work and there is no one to help bring up the children  3 Policies on marriage and childbearing leave, maternity allowance, and employment protection for female workers are not yet in place  4 Inadequate institutions for childcare and care of children aged 0-3 years do not meet the needs of the public  5 Worried that personal career development will be affected  6 Older age and limited energy  7 Opinion of the eldest son or daughter  8 Want to become a Sink family  9 Not yet  10 Other |
| 15. If you evaluate socio-economic status on a scale of 1-10, with 1 being the lowest, 5 being the middle, and 10 being the highest (the higher the score, the higher the socio-economic status), you would say that you are currently in the lowest class.  \|---------\|--------\|---------\|--------\|---------\|--------\|--------\|---------\|---------\|  1 2 3 4 5 6 7 8 9 10 |

**II. Mobility and employment**

| 16. What is the main reason for you to go out to work in the past year?  1 Labor/job 2 Business 3 Family migration (to take care of the elderly/children) 4 Marriage  5 Relocation 6 Relocating to friends/relatives 7 Taking care of the family 8 Others |
| --- |
| 17. How long have you been living in the place where you are working?  1. Less than 1 year 2. 1-2 years 3. 3-5 years 4. 6-10 years 5. More than 10 years |
| 18. What is your local housing situation?  1 Dormitory provided by the organization 2 Renting private housing 3 Public housing provided by the government 4 Self-purchased housing 5 Borrowing from friends and relatives 6 Others |
| 19. In the future, do you intend to stay in the local area?  1 Yes 2 No (skip to Q21) 3 Not sure (skip to Q23) |
| 20. What are the main reasons why you intend to stay in the local area?  1 High income level, more money 2 More room for personal development 3 Good medical protection 4 Family members are used to living in the local area 5 Better education opportunities for children 6 Developed city 7 Convenient life 8 Others |
| 21. If you do not plan to stay in your local area, do you choose to return home or go elsewhere?  1 Return home 2 Somewhere else (skip to Q23) 3 Not sure (skip to Q23) |
| 22. What is the most important reason for you to return home? (Multiple choices allowed)  1 High level of local daily consumption  2 Lack of social security such as pension and unemployment insurance  3 Strong policy support in your hometown  4 More suitable employment opportunities in your hometown  5 Elderly people or children at home need to be taken care of  6 Health reasons such as your own injuries or illnesses  7 Persuasion from your family  8 Current labor intensity  9 Poor labor conditions in your job outside your hometown  10 Discrimination from the locals  11 The former part-time work unit closed down  12 A lot of acquaintances have come back to work  13 Other reasons |
| 23. Are you currently employed?  1 Permanent job 2 Temporary job 3 No job |
| 24. The way you look for a job? (Multiple choice)  1 Introduced by friends/relatives/fellow villagers 2 Labor intermediary 3 Government labor export 4 Job advertisement 5 Graduation allocation or school recommendation 6 Self-employment, no help 7 Other ways |
| 25. In the past year, you probably worked about ____ hour per day, ____ day per week. |
| 26. What kind of labor contract do you have with your current workplace (employer)?  1 Fixed-term 2 Open-ended 3 One-time work assignment 4 Probationary period 5 No labor contract 6 Unknown |
| 27. Is your work exposed to any of the following specific substances or unfavorable environments? (Multiple answers allowed)  1 None 2 Strong noise 3 Dust/dust 4 Toxic gases 5 Fume pollution 6 Hazardous mechanical installations 7 Pesticides/insecticides 8 Dye pollution 9 Heavy metals 10 Other pollution |
| 28. Is work in your workplace depressing?  1 Very depressing 2 More depressing 3 Somewhat depressing 4 Less depressing 5 Not at all depressing |
| 29. Are interpersonal relationships in your workplace oppressive?  1 Very depressing 2 More depressing 3 Somewhat depressing 4 Less depressing 5 Not at all depressing |

**III. Healthy lifestyles and behaviors**

| 30. Do you take care to maintain a reasonable weight?  1 Always 2 Often 3 Sometimes 4 Rarely 5 Never |
| --- |
| 31. Are you careful to control an unhealthy diet that is high in salt and fat?  1 Always 2 Often 3 Sometimes 4 Rarely 5 Never |
| 32. How often do you participate in physical activities of light or moderate intensity?  1 Always 2 Often 3 Sometimes 4 Rarely 5 Never |
| 33. Do you get enough sleep?  1 Always 2 Often 3 Sometimes 4 Rarely 5 Never |
| 34. Do you smoke (including e-cigarettes)?  1 Always 2 Often 3 Sometimes 4 Quit smoking 5 Never smoked |
| 35. Do you drink alcohol?  1 Always 2 Often 3 Sometimes 4 Quit drinking 5 Never drink |
| 36. Do you pay attention to mental stress reduction and self-relaxation?  1 Always 2 Often 3 Sometimes 4 Rarely 5 Never |
| 37. How is your relationship with those around you?  1 Very well 2 Quite well 3 Fairly well 4 Not very well 5 Very poorly |
| 38. Do you feel that you are constantly learning or progressively improving?  1 Always 2 Often 3 Sometimes 4 Rarely 5 Never |
| 39. Do you have regular health check-ups (excluding check-ups due to illness)?  1 Always 2 Often 3 Sometimes 4 Rarely 5 Never |

**IV. Health status and medical care**

| 40. In the past year, how healthy do you think you are?  1 Very healthy 2 Fairly healthy 3 Average 4 Unhealthy 5 Very unhealthy |
| --- |
| 41. Do you currently have a chronic illness that has been diagnosed by a doctor? (Multiple choices allowed)  1 No 2 Hypertension 3 Diabetes 4 Cardiovascular disease (coronary heart disease, etc.)  5 Cerebrovascular disease (stroke, cerebral infarction, cerebral thrombosis, etc.) 6 Chronic Obstructive Pulmonary Disease 7 Cancer 8 Other diseases |
| 42. Do you suffer from any of the following gynecological diseases diagnosed by a doctor (multiple answers allowed)? (Female only)  1 No 2 Uterine fibroid 3 Ovarian cyst 4 Polycystic ovary syndrome 5 Vaginitis 6 Mastitis 7 Pelvic inflammatory disease 8 Breast cancer 9 Cervical cancer 10 Tubal inflammation 11 Endometrial cancer 12 Ovarian cancer 13 Vulvar/vaginal tumor 14 Others |
| 43. During the last two weeks, have you had any illnesses (injuries) or medical conditions?  1 Yes 2 No (skip to Q46) |
| 44. What did you do when you became ill?  1 Self-medicated 2 Did nothing 3 Went to hospital (skip to Q46) |
| 45. Reasons for not seeking medical treatment?  1 Consultation a fortnight ago, continuing treatment as prescribed 2 Self-perceived improvement 3 Financial difficulties 4 Trouble attending 5 No time 6 Transport difficulties 7 No effective measures 8 Other reasons |
| 46. In the past year, when you were sick (injured) or unwell, which institution did you prefer to go to?  1 Workplace community health station (center/street health center) 2 Workplace individual clinic 3 Workplace general/specialist hospital 4 Return to hometown for medical treatment |
| 47. What are the main reasons for your preference for this institution (multiple choices allowed)?  1 This institution has high technical level and good reputation 2 Self-perceived mild (serious) condition 3 Familiar doctors 4 Convenient and quick access to the doctor 5 Reasonable services (including medicines and examinations) 6 Cheap/appropriate price 7 Good service attitude 8 Good environment for consultation 9 High reimbursement rate 10 Others |
| 48. How long did you wait in the queue for your consultation? Minutes |
| 49. In the last year, have you had a doctor's diagnosis that required hospitalization and you were not hospitalized?  1 Yes 2 No (skip to Q51) |
| 50. What are your reasons for not being hospitalized when you needed to be (multiple answers allowed)?  1 Didn't think you needed it 2 Didn't think there were effective treatments available 3 Financial difficulties 4 Thought hospital services were poor 5 Didn't have time for myself 6 No hospital beds available 7 Health insurance restrictions 8 Other |
| 51. Which of the following types of insurance do you have? (Multiple choices allowed)  1 Urban workers' basic medical insurance 2 Urban and rural residents' basic medical insurance (former urban residents' basic medical insurance and new rural medical insurance)  3 Commercial insurance 4 No insurance (skip to Q55) 5 Others |
| 52. Where are you insured?  1 Place of residence 2 Place of employment 3 Both place of residence and place of employment  4 Others |
| 53. Have you ever enjoyed the policy of reimbursement for medical treatment in a different place?  1 Completed the record of medical treatment in a different place and enjoyed the reimbursement  2 Completed the record of medical treatment in a different place but did not enjoy the reimbursement  3 Did not complete the record of medical treatment in a different place  4 Not sure about the policy of reimbursement for medical treatment in a different place |
| 54. How much do you pay for your medical expenses?  1 Reimbursed by health insurance, partially out-of-pocket 2 Fully out-of-pocket 3 Publicly funded medical care 4 Other |
| **Mental Health Measurement: In the past two weeks, how often have the following symptoms occurred in your life: (PHQ-9/GAD-7)** |
| 55. Can't get motivated or uninterested in doing things  1 Not at all 2 Several days 3 More than half the days 4 Almost every day |
| 56. Feeling down, depressed or hopeless  1 Not at all 2 Several days 3 More than half the days 4 Almost every day |
| 57. Difficulty falling asleep, restless sleep, or sleeping too much  1 Not at all 2 Several days 3 More than half the days 4 Almost every day |
| 58. Feeling tired or lack of energy  1 Not at all 2 Several days 3 More than half the days 4 Almost every day |
| 59. Loss of appetite or eating too much  1 Not at all 2 Several days 3 More than half the days 4 Almost every day |
| 60. Feeling bad about myself - or feeling like a failure or letting myself/family down  1 Not at all 2 Several days 3 More than half the days 4 Almost every day |
| 61. has trouble concentrating on things, e.g. when reading the newspaper or watching TV  1 Not at all 2 Several days 3 More than half the days 4 Almost every day |
| 62. Move or speak so slowly that others have noticed? Or just the opposite, irritable or fidgety, moving around more than usual  1 Not at all 2 Several days 3 More than half the days 4 Almost every day |
| 63. Thoughts of being better off dead or hurting myself in some way  1 Not at all 2 Several days 3 More than half the days 4 Almost every day |
| 64. Feeling nervous, anxious or eager  1 Not at all 2 Several days 3 More than half the days 4 Almost every day |
| 65. Not able to stop or control worrying  1 Not at all 2 Several days 3 More than half the days 4 Almost every day |
| 66. Worry too much about various things  1 Not at all 2 Several days 3 More than half of the days 4 Almost every day |
| 67. Difficult to relax  1 not at all 2 several days 3 more than half the days 4 almost every day |
| 68. Unable to sit still because of restlessness  1 Not at all 2 Several days 3 More than half of the days 4 Almost every day |
| 69. Become easily annoyed or impatient  1 Not at all 2 Several days 3 More than half the days 4 Almost every day |
| 70. Feeling scared as if something terrible is going to happen  1 Not at all 2 For several days 3 More than half the days 4 Almost every day |

**V. Health service utilization**

| 71. Have you heard of the National Basic Public Health Service?  1 Yes 2 No |
| --- |
| 72. In the past year, have you received any health education provided by the community health centers (stations)/township health centers at your workplace?  1 Yes 2 No (skip to Q75) |
| 73. In the past year, which of the following aspects of health education did you receive at the community health service center (station)/township health center at your workplace? (Multiple choices allowed)  1 Prevention and treatment of occupational diseases  2 Prevention and treatment of sexually transmitted diseases/AIDS  3 Reproductive health and contraception  4 Prevention and treatment of tuberculosis  5 Smoking control  6 Mental health  7 Prevention and treatment of chronic diseases  8 Maternal and child health care/preferential childbearing  9 Self-help in public emergencies |
| 74. In what way did you receive the above health education at the community health service center (station)/township health center at your workplace? (Multiple choices allowed)  1 Health knowledge lectures and public health consultation activities 2 Promotional materials (paper, film and television) 3 Radio and television 4 Friends and family in the neighborhood 5 Medical staff 6 Community WeChat groups, Jitterbugs and other platforms 7 Others |
| 75. How long does it take to get to the nearest health service provider (including community health centers, village clinics, hospitals, etc.) from your place of residence at the time of your work? (by your most accessible mode of transport)?  1. Under 15 minutes 2. 15 minutes-30 minutes (inclusive)  3. 30 minutes (not included) -1 hour (included) 4. More than 1 hour |
| 76. Have you set up a population health record?  1 Yes, already established 2 No, but heard of it 3 No, never heard of it 4 Not sure |
| 77. In the past year, have you proactively sought access to health care or related assistance from your workplace community?  1 Yes 2 No |
| 78. Have you signed up for family doctor service?  1 Yes, signed up 2 Not signed up, but heard of it 3 Not signed up, never heard of it 4 Not sure |
| 79. Have you received maternal and child health care services such as maternal and child health care guidance and prenatal check-ups at the community health centers (stations)/township health centers in your workplace? (Only women who have already had children answered)  1 Yes 2 No 3 Not sure |
| 80. Have you received free cervical and breast cancer screening at your place of work? (Female respondents only)  1 Received free screening 2 Heard about it but did not receive free screening 3 Never heard of this policy |
