# Supplementary material for: Factors influencing health service utilization among 19,869 China’s migrant population: an empirical study based on the Andersen behavioral model
Source: Front Public Health. 2025 Jan 23;13:1456839. doi: 10.3389/fpubh.2025.1456839 (PMC11798976; doi:10.3389/fpubh.2025.1456839)
Supplement: Supplementary file 2 [file Table_1.docx]

| **Variables** |  | **0 Health service utilization** | | | | | | | | |  | **1-2 Health service utilization** | | | | | | | | |
| --- | --- | --- | --- | --- | --- | --- | --- | --- | --- | --- | --- | --- | --- | --- | --- | --- | --- | --- | --- | --- |
|  |  | ***B*** |  | ***SE*** |  | ***Wald χ2*** |  | ***OR*** |  | ***95% CI*** |  | ***B*** |  | ***SE*** |  | ***Wald χ2*** |  | ***OR*** |  | ***95% CI*** |
| **10-27** |  | 2.571 |  | 0.062 |  | 1717.848** |  | 13.080 |  | 11.583-14.771 |  | 1.284 |  | 0.060 |  | 451.452** |  | 3.610 |  | 3.207-4.063 |
| **28-33** |  | 1.804 |  | 0.060 |  | 903.947** |  | 6.074 |  | 5.400-6.832 |  | 1.043 |  | 0.057 |  | 337.240** |  | 2.837 |  | 2.538-3.171 |
| **34-37** |  | 1.068 |  | 0.059 |  | 329.217** |  | 2.910 |  | 2.593-3.266 |  | 0.706 |  | 0.054 |  | 172.105** |  | 2.025 |  | 1.823-2.251 |
| **38-50** |  |  |  |  |  |  |  | 1 (ref) |  |  |  |  |  |  |  |  |  | 1 (ref) |  |  |

**Table S1.** **Health service utilization of participants with different lifestyle scores (n=19869).**

**p<0.01, *p<0.05
