# Supplementary material for: Factors influencing health service utilization among 19,869 China’s migrant population: an empirical study based on the Andersen behavioral model
Source: Front Public Health. 2025 Jan 23;13:1456839. doi: 10.3389/fpubh.2025.1456839 (PMC11798976; doi:10.3389/fpubh.2025.1456839)
Supplement: Supplementary file 3 [file Table_2.docx]

**Table S2. Multiple logistic regression analysis of health service utilization among participants (n=19869).**

| **Variables** |  | **0 Health service utilization** | | | | | | | | |  | **1-2 Health service utilization** | | | | | | | | |
| --- | --- | --- | --- | --- | --- | --- | --- | --- | --- | --- | --- | --- | --- | --- | --- | --- | --- | --- | --- | --- |
|  |  | ***B*** |  | ***SE*** |  | ***Wald χ2*** |  | ***OR*** |  | ***95% CI*** |  | ***B*** |  | ***SE*** |  | ***Wald χ2*** |  | ***OR*** |  | ***95% CI*** |
| **Predisposing** |  |  |  |  |  |  |  |  |  |  |  |  |  |  |  |  |  |  |  |  |
| **Gender** |  |  |  |  |  |  |  |  |  |  |  |  |  |  |  |  |  |  |  |  |
| Male |  | 0.047 |  | 0.049 |  | 0.908 |  | 1.048 |  | 0.952-1.154 |  | -.048 |  | 0.048 |  | 1.033 |  | 0.953 |  | 0.868-.046 |
| Female |  |  |  |  |  |  |  | 1 (ref) |  |  |  |  |  |  |  |  |  |  |  | 1 (ref) |
| **Age (years)** |  |  |  |  |  |  |  |  |  |  |  |  |  |  |  |  |  |  |  |  |
| ≤ 25 |  | 0.420 |  | 0.135 |  | **9.622**** |  | 1.522 |  | 1.167-1.985 |  | 0.402 |  | 0.130 |  | **9.627**** |  | 1.495 |  | 1.160-1.927 |
| 26-35 |  | 0.705 |  | 0.109 |  | **41.649**** |  | 2.024 |  | 1.634-2.508 |  | 0.462 |  | 0.103 |  | **20.122**** |  | 1.588 |  | 1.297-1.943 |
| 36-45 |  | 0.698 |  | 0.107 |  | **42.791**** |  | 2.009 |  | 1.630-2.476 |  | 0.492 |  | 0.100 |  | **24.127**** |  | 1.636 |  | 1.344-1.991 |
| 46-55 |  | 0.414 |  | 0.110 |  | **14.165**** |  | 1.513 |  | 1.219-1.877 |  | 0.279 |  | 0.103 |  | **7.305**** |  | 1.322 |  | 1.080-1.618 |
| ≥56 |  |  |  |  |  |  |  | 1 (ref) |  |  |  |  |  |  |  |  |  | 1 (ref) |  |  |
| **BMI (kg/m^2^)** |  |  |  |  |  |  |  |  |  |  |  |  |  |  |  |  |  |  |  |  |
| < 18.5 |  | 0.215 |  | 0.118 |  | 3.323 |  | 1.240 |  | 0.984-1.563 |  | 0.205 |  | 0.117 |  | 3.045 |  | 1.227 |  | 0.975-1.545 |
| 18.5-27.9 |  | 0.040 |  | 0.068 |  | 0.354 |  | 1.041 |  | 0.911-1.190 |  | 0.156 |  | 0.069 |  | **5.170*** |  | 1.169 |  | 1.022-1.338 |
| ≥28 |  |  |  |  |  |  |  | 1 (ref) |  |  |  |  |  |  |  |  |  | 1 (ref) |  |  |
| **Ethnicity** |  |  |  |  |  |  |  |  |  |  |  |  |  |  |  |  |  |  |  |  |
| Han |  | 0.130 |  | 0.178 |  | 0.540 |  | 1.139 |  | 0.804-1.614 |  | 0.009 |  | 0.167 |  | 0.003 |  | 1.009 |  | 0.727-1.399 |
| Minorities |  |  |  |  |  |  |  | 1 (ref) |  |  |  |  |  |  |  |  |  | 1 (ref) |  |  |
| **Marital status** |  |  |  |  |  |  |  |  |  |  |  |  |  |  |  |  |  |  |  |  |
| Unmarried |  | 0.017 |  | 0.150 |  | 0.013 |  | 1.017 |  | 0.758-1.365 |  | 0.032 |  | 0.149 |  | 0.045 |  | 1.032 |  | 0.770-1.383 |
| Married |  | -0.059 |  | 0.112 |  | 0.275 |  | 0.943 |  | 0.756-1.175 |  | -0.090 |  | 0.112 |  | 0.644 |  | 0.914 |  | 0.735-1.138 |
| Divorced/Widowed |  |  |  |  |  |  |  | 1 (ref) |  |  |  |  |  |  |  |  |  | 1 (ref) |  |  |
| **Educational level** |  |  |  |  |  |  |  |  |  |  |  |  |  |  |  |  |  |  |  |  |
| Primary and below |  | 0.636 |  | 0.109 |  | **34.164**** |  | 1.889 |  | 1.526-2.338 |  | 0.308 |  | 0.117 |  | **6.942**** |  | 1.361 |  | 1.082-1.711 |
| Junior high school |  | 0.555 |  | 0.047 |  | **136.676**** |  | 1.742 |  | 1.587-1.912 |  | 0.361 |  | 0.050 |  | **52.675**** |  | 1.435 |  | 1.301-1.581 |
| High school |  | -0.166 |  | 0.050 |  | **11.176**** |  | 0.847 |  | 0.769-0.934 |  | -0.108 |  | 0.051 |  | **4.462*** |  | 0.897 |  | 0.812-0.992 |
| University or college degree and above |  |  |  |  |  |  |  | 1 (ref) |  |  |  |  |  |  |  |  |  | 1 (ref) |  |  |
| **Solitary** |  |  |  |  |  |  |  |  |  |  |  |  |  |  |  |  |  |  |  |  |
| No |  | -0.004 |  | 0.045 |  | 0.007 |  | 0.996 |  | 0.913-1.088 |  | -0.062 |  | 0.044 |  | 1.977 |  | 0.940 |  | 0.862-1.025 |
| Yes |  |  |  |  |  |  |  | 1 (ref) |  |  |  |  |  |  |  |  |  | 1 (ref) |  |  |
| **Number of children** |  |  |  |  |  |  |  |  |  |  |  |  |  |  |  |  |  |  |  |  |
| None |  | 0.113 |  | 0.116 |  | 0.958 |  | 1.120 |  | 0.893-1.405 |  | 0.053 |  | 0.116 |  | 0.207 |  | 1.054 |  | 0.840-1.323 |
| 1 |  | -0.108 |  | 0.080 |  | 1.837 |  | 0.897 |  | 0.767-1.049 |  | 0.004 |  | 0.080 |  | 0.002 |  | 1.004 |  | 0.859-1.173 |
| 2 |  | -0.135 |  | 0.075 |  | 3.284 |  | 0.873 |  | 0.755-1.011 |  | -0.009 |  | 0.075 |  | 0.014 |  | 0.991 |  | 0.856-1.147 |
| 3 and above |  |  |  |  |  |  |  | 1 (ref) |  |  |  |  |  |  |  |  |  | 1 (ref) |  |  |
| **Enabling** |  |  |  |  |  |  |  |  |  |  |  |  |  |  |  |  |  |  |  |  |
| **Incoming monthly (RMB)** |  |  |  |  |  |  |  |  |  |  |  |  |  |  |  |  |  |  |  |  |
| <3000 |  | -0.360 |  | 0.064 |  | **31.200**** |  | 0.698 |  | 0.615-0.792 |  | -0.237 |  | 0.063 |  | **14.080**** |  | 0.789 |  | 0.698-0.893 |
| 3000-4999 |  | -0.276 |  | 0.048 |  | **32.367**** |  | 0.759 |  | 0.690-0.835 |  | -0.146 |  | 0.048 |  | **9.349**** |  | 0.864 |  | 0.787-0.949 |
| ≥5000 |  |  |  |  |  |  |  | 1 (ref) |  |  |  |  |  |  |  |  |  | 1 (ref) |  |  |
| **Employment status** |  |  |  |  |  |  |  |  |  |  |  |  |  |  |  |  |  |  |  |  |
| Permanent work |  | -0.198 |  | 0.063 |  | **9.982**** |  | 0.820 |  | 0.726-0.928 |  | -0.110 |  | 0.066 |  | 2.802 |  | 0.896 |  | 0.788-1.019 |
| Temporary work |  | -0.128 |  | 0.062 |  | **4.255*** |  | 0.880 |  | 0.779-0.994 |  | -0.111 |  | 0.065 |  | 2.901 |  | 0.895 |  | 0.787-1.017 |
| None |  |  |  |  |  |  |  | 1 (ref) |  |  |  |  |  |  |  |  |  | 1 (ref) |  |  |
| **Housing condition** |  |  |  |  |  |  |  |  |  |  |  |  |  |  |  |  |  |  |  |  |
| Rent |  | 0.138 |  | 0.074 |  | 3.510 |  | 1.148 |  | 0.994-1.327 |  | 0.189 |  | 0.072 |  | **6.923**** |  | 1.208 |  | 1.049-1.391 |
| Own house |  |  |  |  |  |  |  | 1 (ref) |  |  |  |  |  |  |  |  |  | 1 (ref) |  |  |
| **Insurance status** |  |  |  |  |  |  |  |  |  |  |  |  |  |  |  |  |  |  |  |  |
| No |  | 0.665 |  | 0.083 |  | **64.233**** |  | 1.944 |  | 1.653-2.288 |  | 0.437 |  | 0.084 |  | **26.817**** |  | 1.547 |  | 1.312-1.825 |
| Yes |  |  |  |  |  |  |  | 1 (ref) |  |  |  |  |  |  |  |  |  | 1 (ref) |  |  |
| **Distance from residence to nearest medical institution** |  |  |  |  |  |  |  |  |  |  |  |  |  |  |  |  |  |  |  |  |
| < 15min |  | -1.584 |  | 0.133 |  | **142.720**** |  | 0.205 |  | 0.158-0.266 |  | -0.844 |  | 0.138 |  | **37.316**** |  | 0.430 |  | 0.328-0.564 |
| 15-30min |  | -1.013 |  | 0.134 |  | **56.798**** |  | 0.363 |  | 0.279-0.473 |  | -0.416 |  | 0.140 |  | **8.830**** |  | 0.660 |  | 0.501-0.868 |
| 31-60min |  | -0.492 |  | 0.149 |  | **10.899**** |  | 0.611 |  | 0.456-0.819 |  | -0.192 |  | 0.155 |  | 1.525 |  | 0.825 |  | 0.609-1.119 |
| > 60min |  |  |  |  |  |  |  | 1 (ref) |  |  |  |  |  |  |  |  |  | 1 (ref) |  |  |
| **SES** |  |  |  |  |  |  |  |  |  |  |  |  |  |  |  |  |  |  |  |  |
| Low |  | 0.901 |  | 0.110 |  | **66.548**** |  | 2.461 |  | 1.982-3.055 |  | 0.385 |  | 0.098 |  | **15.440**** |  | 1.470 |  | 1.213-1.781 |
| Moderate |  | 0.332 |  | 0.113 |  | **8.624**** |  | 1.394 |  | 1.117-1.741 |  | 0.227 |  | 0.100 |  | **5.177*** |  | 1.255 |  | 1.032-1.527 |
| High |  |  |  |  |  |  |  | 1 (ref) |  |  |  |  |  |  |  |  |  | 1 (ref) |  |  |
| **Mental health status** |  |  |  |  |  |  |  |  |  |  |  |  |  |  |  |  |  |  |  |  |
| Severe |  | 1.187 |  | 0.193 |  | **37.689**** |  | 3.276 |  | 2.243-4.785 |  | 0.528 |  | 0.204 |  | **6.689*** |  | 1.695 |  | 1.136-2.529 |
| Moderately severe |  | 0.688 |  | 0.108 |  | **40.567**** |  | 1.989 |  | 1.610-2.458 |  | 0.336 |  | 0.112 |  | **8.939**** |  | 1.399 |  | 1.123-1.743 |
| Moderate |  | 0.760 |  | 0.084 |  | **82.756**** |  | 2.139 |  | 1.816-2.520 |  | 0.454 |  | 0.086 |  | **28.128**** |  | 1.575 |  | 1.332-1.863 |
| Mild |  | 0.450 |  | 0.054 |  | **69.089**** |  | 1.568 |  | 1.410-1.744 |  | 0.242 |  | 0.054 |  | **19.837**** |  | 1.273 |  | 1.145-1.416 |
| Normal |  |  |  |  |  |  |  | 1 (ref) |  |  |  |  |  |  |  |  |  | 1 (ref) |  |  |
| **Need** |  |  |  |  |  |  |  |  |  |  |  |  |  |  |  |  |  |  |  |  |
| **Having chronic disease** |  |  |  |  |  |  |  |  |  |  |  |  |  |  |  |  |  |  |  |  |
| No |  | 0.019 |  | 0.062 |  | 0.091 |  | 1.019 |  | 0.902-1.152 |  | 0.002 |  | 0.062 |  | 0.001 |  | 1.002 |  | 0.887-1.132 |
| Yes |  |  |  |  |  |  |  | 1 (ref) |  |  |  |  |  |  |  |  |  | 1 (ref) |  |  |
| **Self-evaluation general health status** |  |  |  |  |  |  |  |  |  |  |  |  |  |  |  |  |  |  |  |  |
| Unhealthy |  | 0.818 |  | 0.136 |  | **36.164**** |  | 2.267 |  | 1.736-2.960 |  | 0.617 |  | 0.140 |  | **19.480**** |  | 1.853 |  | 1.409-2.436 |
| Moderate |  | 0.296 |  | 0.052 |  | **32.193**** |  | 1.344 |  | 1.214-1.489 |  | 0.129 |  | 0.053 |  | **5.986*** |  | 1.138 |  | 1.026-1.262 |
| Healthy |  |  |  |  |  |  |  | 1 (ref) |  |  |  |  |  |  |  |  |  | 1 (ref) |  |  |
| **Illness in the last two weeks** |  |  |  |  |  |  |  |  |  |  |  |  |  |  |  |  |  |  |  |  |
| No |  | 0.058 |  | 0.075 |  | 0.613 |  | 1.060 |  | 0.916-1.227 |  | 0.156 |  | 0.075 |  | **4.338*** |  | 1.169 |  | 1.009-1.354 |
| Yes |  |  |  |  |  |  |  | 1 (ref) |  |  |  |  |  |  |  |  |  | 1 (ref) |  |  |
| **Requires hospitalization not hospitalized** |  |  |  |  |  |  |  |  |  |  |  |  |  |  |  |  |  |  |  |  |
| No |  | 1.378 |  | 0.080 |  | **296.424**** |  | 3.966 |  | 3.390-4.640 |  | 0.909 |  | 0.076 |  | **141.746**** |  | 2.481 |  | 2.136-2.881 |
| Yes |  |  |  |  |  |  |  | 1 (ref) |  |  |  |  |  |  |  |  |  | 1 (ref) |  |  |

**p<0.01, *p<0.05
