# Supplementary material for: Factors influencing health service utilization among 19,869 China’s migrant population: an empirical study based on the Andersen behavioral model
Source: Front Public Health. 2025 Jan 23;13:1456839. doi: 10.3389/fpubh.2025.1456839 (PMC11798976; doi:10.3389/fpubh.2025.1456839)
Supplement: Supplementary file 4 [file Table_3.docx]

**Table S3. The list of variables for empirical analysis.**

| **Predisposing factors** |  | Demography |  | Age |  | ≤25; 26-35; 36-45; 46-55; >56 |
| --- | --- | --- | --- | --- | --- | --- |
|  |  |  |  | Gender |  | Male; Female |
|  |  | Social structure |  | Educational level |  | Primary and below; Junior high school; High school; University or college degree and above |
|  |  |  |  | Ethnicity |  | Han ethnic; Minorities |
|  |  |  |  | Marital status |  | Unmarried; married; divorced/widowed |
|  |  |  |  | BMI |  | < 18.5; 18.5-27.9; ≥28 |
|  |  |  |  | Solitary |  | Yes; No |
|  |  |  |  | Number of children |  | None; 1; 2; 3 and above |
| **Enabling factors** |  | Individual / family resources |  | Incoming monthly |  | <3000; 3000-4999; ≥5000 |
|  |  |  |  | Employment status |  | Permanent work; Temporary work; None |
|  |  |  |  | Insurance status |  | Yes; No |
|  |  |  |  | Housing condition |  | Own house; Rent |
|  |  |  |  | Mental health status |  | Normal; Mild; Moderately; Moderately severe; Severe |
|  |  |  |  | SES |  | Low; Moderate; High |
|  |  |  |  | Healthy lifestyle score |  | Q1; Q2; Q3; Q4 |
|  |  | Community resources |  | Distance from residence to nearest medical institution |  | < 15min; 15-30min; 31-60min; > 60min |
| **Needing factors** |  | Having chronic disease |  |  |  | Yes; No |
|  |  | Self-evaluation general health status |  |  |  | Healthy; General; Unhealthy |
|  |  | Illness in the last two weeks |  |  |  | Yes; No |
|  |  | Needed but not hospitalized |  |  |  | Yes; No |
| **Health service utilization indicators** |  | Family doctor |  |  |  | Yes; No |
|  |  | Health education |  |  |  | Yes; No |
|  |  | Proactively seeking health care knowledge |  |  |  | Yes; No |
|  |  | Health profile |  |  |  | Yes; No |
